# Treatment of cerebral ventriculitis with a new self-irrigating catheter system: narrative review and case series

**DOI:** 10.1186/s44158-023-00131-5

**Published:** 2023-11-08

**Authors:** Gloria Stati, Ernesto Migliorino, Manuel Moneti, Carlo Alberto Castioni, Antonino Scibilia, Giorgio Palandri, Giulio Virgili, Raffaele Aspide

**Affiliations:** 1 Anesthesia and Intensive Care School, University of Bologna, Resident, Bologna, Italy; 2 IRCCS Istituto delle Scienze Neurologiche di Bologna, Anesthesia and Intensive Care Unit, Bologna, Italy; 3 IRCCS Istituto delle Scienze Neurologiche di Bologna, Neurosurgery Unit, Bologna, Italy; 4 Department for Integrated Infectious Risk Management, AUSL of Bologna-S. Orsola-Malpighi Hospital, Bologna, Italy

**Keywords:** Cerebral ventriculitis, IRRAflow, Cerebrospinal fluid, Intracranial pressure, External ventricular drain

## Abstract

Cerebral ventriculitis is a life-threatening condition that requires prompt and effective pharmacological intervention. The continuous irrigation of the cerebral ventricles with fluid and its drainage is a system to remove toxic substances and infectious residues in the ventricles; this system is called IRRAflow®. We used this kind of ventricular irrigation/drainage system to treat two patients with post-surgical cerebral ventriculitis and a patient with bacterial meningitis complicated with ventriculitis. In this case series, we discuss the management of these three cases of cerebral ventriculitis: we monitored cytochemical parameters and cultures of the cerebrospinal fluid of patients during their ICU stay and we observed a marked improvement after irrigation and drainage with IRRAflow® system. Irrigation/drainage catheter stay, mode settings, and antibiotic therapies were different among these three patients, and neurological outcomes were variable, according to their underlying pathologies. IRRAflow® system can be applied also in other types of brain injury, such as intraventricular hemorrhage, intracranial abscess, subdural hematomas, and intracerebral hemorrhage, with the aim to remove the hematic residues and enhance the functional recovery of the patients. IRRAflow® seems a promising and useful tool to treat infectious and hemorrhagic diseases in neuro-intensive care unit.

## Introduction

Acute brain injury are events that can cause acute hydrocephalus and the insertion of an external ventricular drain (EVD) has a dual benefit: it allows to drain of cerebrospinal fluid (CSF) and to monitor intracranial pressure (ICP) [1]. The most common complications of EVD placement are new onset hemorrhage, misplacement, and infection the risk of EVDs-related ventriculo-meningitis is 10–17% [2] and its occurrence is favored by the number of manipulations, repositioning, and the total number of days it remains on site [3]. Ventriculitis is also a possible complication of community meningitis [4]. One of the possible treatments for these infections is intraventricular antibiotic administration, however, several aspects are yet to be defined, like treatment dosing and duration and the optimal patient population to treat [5].

**Fig. 1:**
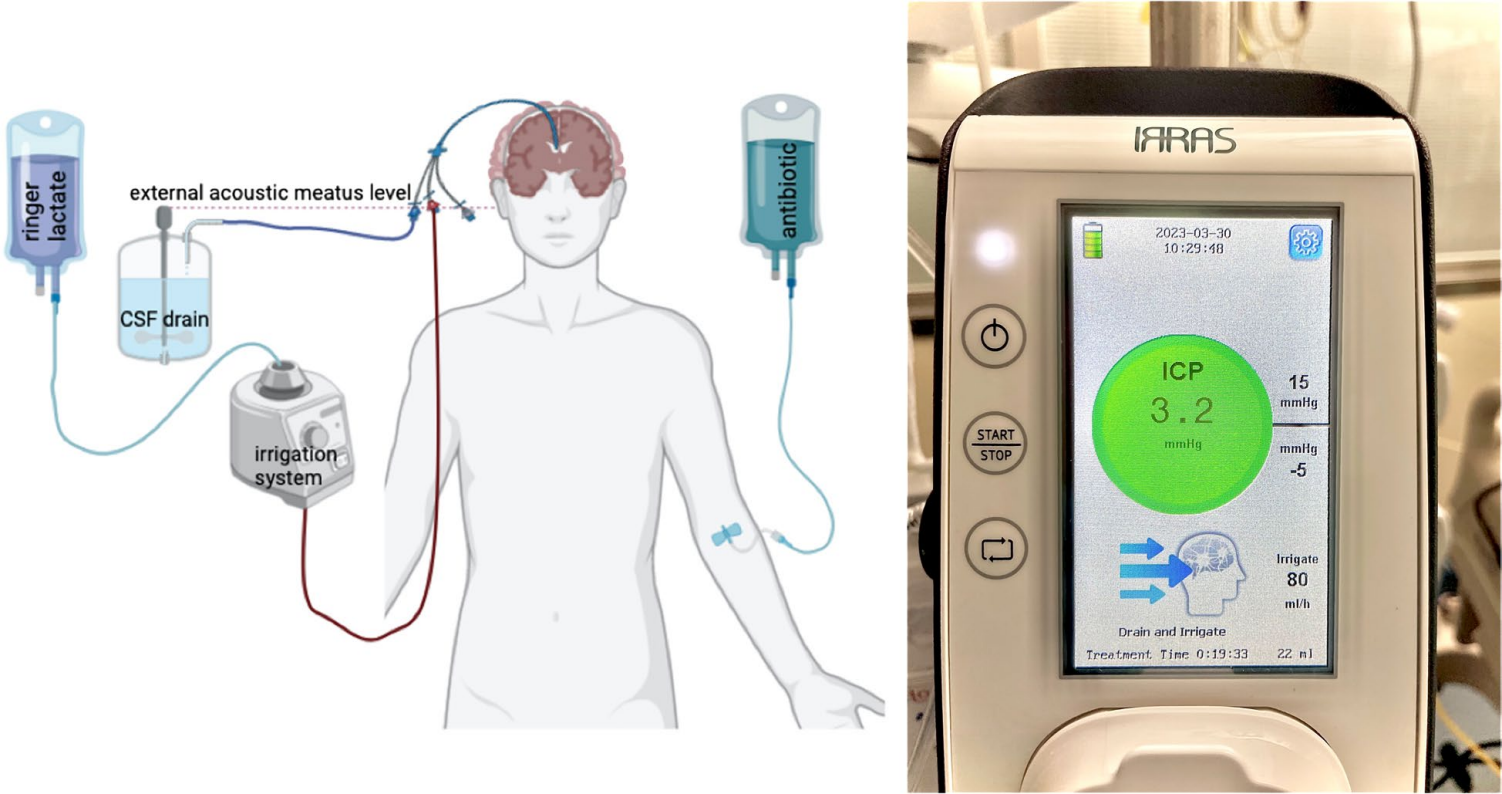
IRRAflow® system: it consists of an irrigating digital pump and a drainage mechanism connected to a dual-lumen catheter. Every cycle has 3 phases: 1. Irrigation, 2. Drainage, 3. Intracranial pressure (ICP) measurement. The cycle automatically stops when the ICP detected is over or under a safety range that can be customized for each patient. Illustration added was created using Biorender®

IRRAflow® (IRRAS, Stockholm, Sweden), a new self-irrigating catheter system, fits into this context. The difference from a standard EVD is that IRRAflow® can drain and inject a predetermined amount of fluid through a dual-lumen catheter (Fig. 1). The difference between this technique and simple EVD is continuous cycle irrigation versus simple gravity and pressure drainage, thus reducing the risk of obstruction and infection because the clots and bacterial colonies are continuously washed away [6]. Initial studies concerned IRRAflow® use to treat chronic subdural hematomas and intraventricular hemorrhage [7] followed by studies regarding the treatment of ventriculitis [8] and intracranial abscess [9].

In this manuscript, the authors present three case reports of patients treated with continuous intraventricular irrigation for ventriculitis using the IRRAflow system without intrathecal antibiotic administration. Finally, the Authors conducted a narrative review of the available literature regarding the different applications of IRRAflow.

## Patients and methods

### Patients, ethics, and consent

The clinical data of three patients, two male and one female, hospitalized in IRCCS Istituto delle Scienze Neurologiche di Bologna, Anesthesia and Intensive Care Unit, Bologna, Italy, were retrospectively reviewed. Patients’ data were anonymized in a database. Written informed consent was obtained from all patients for data treatment.

### Literature analysis

We collected through a review of PUBMED literature from the year 2010 to the present, of articles only in English using as keywords: search#1 [IRRAflow (title/abstract)] AND [External Ventricular drain OR EVD (title/abstract)]; search#2 [IRRAflow (title/abstract)] AND [ventruculitis]. 11 full articles were found, Authors read the abstracts and selected those relevant to the narrative review topic. Nine publications were selected, and reference lists were screened for additional relevant studies.

## Case series

### Case 1

This is a 38 y/o male patient, with a diagnosis of craniopharyngioma. The patient was admitted to the Intensive Care Unit (ICU) for postoperative monitoring after trans-sphenoidal surgery. The patient was extubated on the first postoperative day but subsequently had to be re-intubated due to surgery-related complications. On the fourth day, the patient developed a fever of up to 38.8 °C, probably attributable to pneumonia. Klebsiella pneumoniae was isolated in bronchial lavage fluid and intravenous (IV) targeted-antibiotic therapy was administered. On the eleventh day, a tracheostomy was performed and sedation was interrupted, the patient was quiet and able to voluntarily activate his extremities with a right upper limb plegia. Despite improvement in the patient’s respiratory performance, he developed agitation, a stiff neck, and a deviated gaze. CSF assessment through a lumbar puncture (LP) showed high inflammatory markers, positive for Klebsiella pneumoniae sensitive to antibiotics. Antibiotic therapy was escalated to meropenem and piperacillin/tazobactam at first and then cefotaxime plus levofloxacin, supported by an antibiogram. Magnetic resonance imaging (MRI) demonstrated diffuse enhancement in the III and IV ventricles and signs of intraventricular purulent material, so a ventriculitis diagnosis was declared. The patient developed rhino-liquorrhea due to a CSF fistula, so the patient underwent “redo” surgery and an IRRAflow catheter system was inserted. Irrigation with Lactated Ringer (LR), considered safer in neurosurgery than normal saline and drainage was started the same day. After three days of treatment, CSF was still turbid but CSF cultures were negative and the CSF profile showed a cytochemical improvement. After 6 days of IRRAflow treatment, a CSF leak around the catheter entry site was found and the fluid amount irrigated by IRRAflow was higher than the drained one. The same day IRRAflow catheter was clamped, and a brain Computed Tomography (CT) scan was performed. Imaging showed that the catheter tip leaned against the ventricle wall so, IRRAflow level was raised up to 10 cm from the external acoustic meatus, ventricular volume increased and IRRAflow drain-only mode (without irrigation) restarted. The next day, the IRRAflow system was removed. In the following days the patient’s neurological status and CSF profile showed continuous improvement, and MRI demonstrated a sharp reduction of the previously found ventricular enhancement. Antibiotic therapy was interrupted after 15 days and, on day 30 the patient was discharged to the Neurosurgical ward, with a good neurological status: Glasgow Coma Scale (GCS) 11/15, conditioned by the presence of tracheostomy.

### Case 2

This is a 38-year-old female patient, with a past medical history (PMH) of morbid obesity and Lobstein Syndrome, admitted through the emergency department (ED) after a sudden loss of consciousness (GCS 8/15 and deviated gaze). In the ED she underwent endotracheal intubation, the brain CT scan showed a subarachnoid hemorrhage (SAH) with tetra-ventricular hydrocephalus and intraparenchymal hematoma, caused by a right carotid siphon aneurysm rupture. EVD and a Codman™ ICP monitoring catheter were placed and endovascular coil embolization of the aneurysm was performed. During the embolization procedure, rebleeding occurred, and a second EVD was placed. The patient was admitted to the ICU with physiological value ICP values. Administration of urokinase through both catheters was performed periodically to avoid their occlusion. After gradual sedation weaning, the patient’s neurological examination findings were extremely poor, pupils were reactive, but only a minimal motor response to noxious stimuli was reported (GCS 4/15). The next day tracheostomy was performed and the patient was weaned off by mechanical ventilation. By the following days, neurological status showed a progressive improvement with spontaneous movements of the four limbs and opening of the eyes, appearing conscious but not oriented (GCS 11/15). On day 11, the right-sided EVD catheter stopped draining, brain CT scan was performed, showing displacement of the right catheter and a progressive reduction in ventricular volume, right catheter was removed. In the next days, the patient developed a high fever of up to 39 °C, and blood and CSF samples were cultured. Empirical IV antibiotic therapy was initiated with meropenem. Fever was unresponsive to first-line antibiotic treatment and the patient developed a progressive increase in ICP value, brain CT scan was performed showing a new increase in ventricular size, linezolid was added to the antibiotic therapy and the left-sided EVD catheter was replaced with a new one. In the interim blood and CSF cultures tested positive for Pseudomonas aeruginosa, and linezolid was replaced with fosfomycin. A brain MRI scan showed ventriculitis signs, so the left-sided EVD catheter was replaced with an IRRAflow catheter, and irrigation with LR and drainage were started. Over the subsequent week new blood and CSF sample cultures were drawn and proved negative, inflammatory markers values dropped down and neurological status improved, the patient opened her eyes and showed purposeless movements of the limbs. After 13 days of IRRAflow treatment, another brain MRI scan showed a further increase in right lateral ventricular size, so irrigation by IRRAflow catheter was stopped. A non-communicant hydrocephalus was suspected, ventriculo-cisternostomy was performed and a new IRRAflow right-sided catheter was placed. At this point, the patient had two IRRAflow catheters but the left-sided one was used in drain-only mode. A further brain CT scan showed persistent particulate matter in the ventricular system, drainage and irrigation were re-started using the right IRRAflow catheter. After another week the IRRAflow system was stopped and removed, and the patient underwent right ventriculo-peritoneal shunt placement. In the following days, there was no benefit, neurological status worsened and physicians proceeded with comfort care measures. The patient subsequently died several days later.

### Case 3

This is a 61-year-old male patient affected by human immunodeficiency virus infection, chronic HCV-related hepatitis, and previous cerebral ischemia. The patient was found in a drowsy state, with vomiting and fever, he was brought to ED where he soon developed seizures, treated by IV diazepam. In recent clinical history was reported the presence of a dental abscess in the previous days that had been treated with amoxicillin/clavulanic acid. An emergent brain CT scan was obtained, negative for hemorrhagic events. The tox panel was performed, positive for cocaine, opiates, and methadone. He was transferred to the internal medicine ward; an infectious consultation was requested and empiric antibiotic therapy with cefotaxime and levofloxacin was started. An LP was performed which evidenced turbid CSF and a microbiological exam resulted positive for Streptococcus pneumoniae; also, blood cultures were positive for the same microbial agent; a diagnosis of bacterial meningitis was done. Neurological worsening occurred so the patient was intubated and admitted to the ICU. A brain MRI on day 3 showed contrast enhancement of cortical and cerebellar leptomeninges and signs of intraventricular infection. In order to increase the penetration capacity of the antibiotic into the central nervous system, levofloxacin was changed with fosfomycin. A new LP was performed on day 5, with cytochemical signs of bacterial infection, but no bacterial agent was identified. A brain CT scan confirmed the presence of ventriculitis, so an IRRAflow catheter was placed. Intraventricular irrigation with LR and drainage started on day 6; the patient was extubated the same day. On day 7 IRRAflow drainage got stuck, so an intrathecal urokinase administration was performed, with the resumption of the drainage. A new intrathecal treatment with urokinase administration was performed on day 8. CSF drainage remained poor for the subsequent 24 h, CT scan did not show hydrocephalus or new lesions, so the IRRAflow catheter was removed on day 10. A new LP was performed on day 11 which showed great improvement in CSF cytochemical parameters. Patient was discharged on day 11 with good status: without any neurological deficit. A brain MRI was performed after ICU discharge with evidence of reduction of the endo-ventricular signs of infection.

## Results

The three patients’ CSF cytochemical parameters and culture results before and after IRRAflow treatment are summarized in Table 1. Pre- and post-treatment laboratory data seems to show a “non-inferiority” of the without antibiotic irrigation technique compared to intrathecal and intravenous combined antibiotic therapies.

**Table 1:** Cerebrospinal fluid analysis before and after IRRAflow® treatment

	Pre- IRRAflow®	Post- IRRAflow®
Case 1		
Appearance	Turbid	Opalescent
Protein (mg/dL)	234	1012
Glucose (mg/dL)	22	79
Glucose CSF/serum ratio	< 0.4	0.6
WCC (/mmc)	28,721	195
RBC (/mmc)	47,000	2000
Bacterial culture	Positive	Negative
Case 2		
Appearance	Turbid	Xanthochromic
Protein (mg/dL)	155	79
Glucose (mg/dL)	47	60
Glucose CSF/serum ratio	< 0.4	0.6
WCC (/mmc)	5046	254
RBC (/mmc)	4000	4000
Bacterial culture	Positive	Negative
Case 3		
Appearance	Turbid	Opalescent
Protein (mg/dL)	498	55
Glucose (mg/dL)	1	48
Glucose CSF/serum ratio	< 0.4	0.6
WCC (/mmc)	2661	22
RBC (/mmc)	1000	0
Bacterial culture	Positive	Negative

## Discussion

### IRRAflow® and intraventricular hemorrhage

Intraventricular hemorrhage (IVH) is a dramatic event characterized by high morbidity and mortality rate. There are about 22,000 cases of IVH per year in the USA [10]. Mortality ranges from 67% (traumatic IVH) [11] to 19% (IVH secondary to SAH) [12], IVH caused by a spontaneous ICH has a 30-day mortality rate of 43% [13]. The first-line surgical treatment for spontaneous IVH is the positioning of an EVD. It has been shown that the placement of an EDV significantly reduces the risk of death in the case of IVH especially when it is associated with SAH and ICH [14]. However, 19% of positioned EDVs require at least one repositioning and 45% develop at least one occlusion. When EDV becomes occluded, sterile saline flushings are performed, it is usually necessary to repeat the procedure more than once, and if it fails in unblocking the catheter, it must be replaced. These procedures in their turn can cause complications such as edema around the catheter, the onset of new bleeding, and even the onset of iatrogenic infections [15]. Another method often used in case of occlusion of EDV is the administration of fibrinolytic agents through the catheter in order to restore the drainage [16]. IRRAflow was first introduced in the treatment of IVHs to address these complications related to catheter malfunction, at the moment in fact IRRAflow has a rate of occlusion and replacement ranging from 1 to 7% against 19% of standard catheters [6]. In addition, the DRIFT (drainage, irrigation, fibrinolytic therapy) approach has been developed. This approach involves three actions: drainage, irrigation, and fibrinolytic therapy. The ventricular irrigation is performed with an artificial fibrinolytic solution followed by drainage until the blood residues are radiologically removed. The purpose is to defuse the inflammatory cascade caused by the blood material present in the ventricles which is thought to be the cause of negative cognitive outcome [17]. The DRIFT first application on an adult subject was performed in 2021 with the use of the IRRAflow device. In this case, the use of IRRAflow avoided complications related to standard EVD, showed a clear superiority in ventricular clearance, and had also been shown to be easier to use for administering intraventricular fibrinolytic agents [18]. Currently, there is a phase 2, multicenter, randomized controlled trial on the effectiveness and safety of the use of active irrigation in the EVD (arm-IRRAflow intervention) compared to passive external ventricular drainage (control arm-EVD) [19].

### IRRAflow® and intracranial abscess

Intracranial abscess incidence rate is 1500–2500 cases per year in the USA [20]. Mortality ranges from 50 to 20% [21]. The first line of treatment is broad-spectrum antibiotic therapy which should be modified with a targeted therapy. Surgical options include needle aspiration through a burr hole and open craniotomy for excision [22]. Until now there is only one case in literature that describes the use of IRRAflow to treat an intracranial abscess. The abscess was first treated with a craniotomy to allow the abscess drainage. In this case, the IRRAflow system was implanted with two targets: irrigation with crystalloids and intra-thecal administration of antibiotics. Finally, an IV antibiotic therapy was administered. The IRRAflow irrigation solution was at first 0.9 normal saline and on day 2 vancomycin was added (50 mg in 1 L of normal saline), the irrigation velocity was 20 mL/h. The drainage was removed on day 9. MRI demonstrated the resolution of the abscess and no other neurosurgical issues occurred. The authors declared this approach safe and effective with the future target to treat the abscess only using IRRAflow drainage, instead of open craniotomy, to make a less invasive treatment [9].

### IRRAflow® and chronic subdural hematoma

Chronic subdural hematoma (cSDH) incidence rate is 1.72 per 100,000 persons per year with a higher frequency in elderly subjects. cSDH first-line treatment is burr hole evacuation or craniotomy if the cSDH is multi-loculated [23]. The main problem is the high recurrence rate of this pathology, a sub-galeal or subdural drain after surgery can reduce this risk. In 1999 a study demonstrated a 2.6% recurrence rate in patients with postoperative continuous subdural irrigation and drainage vs a 23.8% recurrence rate in patients treated only with subdural passive drainage (SDD) [24]. A second study in 2017 confirmed the superiority of this technique [25]. In this context, the first use of IRRAflow in a patient with cSDH was made in 2019. The hematoma was evacuated with a mini-craniotomy and the IRRAflow irrigating drain was placed in subdural space. The irrigation started at a rate of 10 ml/h with normal saline. In postoperative day 2 the IRRAflow drain was removed, and the patient was discharged the day after with a complete resolution of the midline shift and minimal extracranial fluid collection. On day 14, the patient demonstrated a total resolution of the symptoms [26]. After this case, another patient with recurrent cSDH was treated with middle meningeal artery (MMA) embolization followed by surgical drainage of the hematoma using IRRAflow. The study demonstrated that the combination of burr hole, IRRAflow placement, and MMA embolization was safe and effective with a length of stay of 4.5 days rather than 6 days in the case of standard surgical treatment [7]. In conclusion, in 2021 a retrospective case analysis about the use of craniotomy followed by irrigation with the IRRAflow system in patients with cSDH was made. The study involved 6 patients and demonstrated a significant cSDH recurrence rate reduction (9.3% vs. 24%), a decreased length of stay (2,67 vs. 6.0), lower incidence of postoperative bleeds, infections, seizures, and no catheter misplacement vs 17% in case of SDD. The theory that could explain the excellent effectiveness of this technique is that irrigation promotes evacuation of residual blood and drainage creates a negative gradient that improves brain re-expansion minimizing the postoperative incidence of pneumo-encephalon, recurrent hematoma, and new blood collection [27].

### IRRAflow® and cerebral intraparenchymal hemorrhage

ICH is a terrible event that occurs in 6.5–19.6% of cases of stroke [28]. Mortality is high, survival rate is 40% at 1 year and 24% at 10 years [29]. The management is both medical and surgical. Surgical treatment focuses on surgical decompression in case of intracranial hypertension and hematoma evacuation [30]. In this context, a minimally invasive approach is becoming more feasible. This technique includes a burr hole followed by the insertion of a catheter to drain the hematoma. A 2012 meta-analysis showed the superiority of a minimally invasive approach in supratentorial ICH in terms of mortality and recovery [31]. These results were confirmed in 2018 when a second meta-analysis involving 2466 patients with supratentorial ICH demonstrated lower mortality and rebleeding rates and a higher rate of good recovery in patients treated by minimally invasive approach [32]. Minimally Invasive Surgery Plus recombinant tissue Plasminogen Activator (rtPA) in Intracerebral Haemorrhage Evacuation (MISTIE) approach is an innovative technique that includes the minimally invasive approach described above plus a rtPA administration into the hematoma. For now, MISTIE cannot be considered as a routine care in supratentorial ICH [33], but a MISTIE phase 2 study demonstrated the importance of an ideal catheter to achieve optimal results with this technique [34]. In 2022 the first application of IRRAflow in ICH was made. The study reported 2 patients with basal ganglia ICH treated with the MISTIE approach by using the IRRAflow irrigation system. The result was a successful volume reduction in both patients’ hematomas [35].

### IRRAflow® and ventriculitis

Ventriculitis incidence rate is not clear, the available data mainly concern ventriculitis secondary to meningitis and EVD-related ventriculitis. EVD-related Ventriculitis has an incidence rate that ranges from 0 to 45% [2]. Until now there is only one case in literature that describes the use of IRRAflow to treat ventriculitis. The patient was also treated with IV antibiotic therapy. At first, IRRAflow was set to drain-only mode, it soon became clogged and a second IRRAflow catheter was placed. On day 2 irrigation with normal saline was started by setting both IRRAflow catheters at a velocity of 40 ml/h. In a subsequent step, vancomycin was added and velocity was increased to 60 ml/h. After 3 days of IRRAflow treatment CSF profile, inflammatory markers, and MRI images improved. Despite this, the patient’s clinical status progressively deteriorated and the family asked to proceed with comfort care measures [8]. The use of IRRAflow in ventriculitis treatment has several advantages: its aggressive continued irrigation helps to clear ventricles from pus and inflammatory bioproducts, thus reducing the risk of scarring of the ependymal surface that can ultimately lead to septation and loculated hydrocephalus [36]. Anyway, there is no clear consensus on the use of intrathecal antibiotic therapy to treat ventriculitis due to the risk of seizures and aseptic meningitis [37].

## Limitations

There are several limitations in this study. First of all, the small number of cases included does not allow us to extrapolate any statistically significant conclusions. Furthermore, the absence of randomization precludes the possibility of certain statements. Finally, intrinsic limits to the technique have appeared. Trained nurses and intensive care monitoring are fundamental for the safe use of the IRRAflow system. The irrigation rate and the ICP range can be chosen, but the drainage depends only on the height at which the system is positioned relative to the patient. For this reason, it is necessary to strictly monitor the amount of drainage to avoid the risk of hydrocephalus.

## Conclusions

The use of an IRRAflow catheter for continuous intraventricular irrigation with LR in ventriculitis treatment shows a clear improvement in CSF profiles and imaging findings. More research is needed to define some important aspects like the best velocity rate, the best choice between normal saline and Lactate Ringer, CSF dosage of the antibiotic administered intravenously, and finally when it is safe and recommended the addiction of intrathecal antibiotic therapy.

## Data Availability

The data supporting this study’s findings are available from the corresponding author, GS, upon reasonable request.

## Abbreviations

EVD: External ventricular drains
CSF: Cerebrospinal fluid
ICP: Intracranial pressure
PMH: Past medical history
ICU: Intensive care unit
IV: Intravenous
LP: Lumbar puncture
MRI: Magnetic resonance imaging
LR: Lactate Ringer
CT: Computer tomography
ED: Emergency department
SAH: Subarachnoid hemorrhage
rtPA: Recombinant tissue plasminogen activator
IVH: Intraventricular hemorrhage
ICH: Intracerebral hemorrhage
cSDH: Chronic subdural hematoma
SDD: Subdural passive drainage
MMA: Middle meningeal artery
MISTIE: Minimally invasive surgery plus rtPA in intracerebral haemorrhage evacuation
